# Detection of DNA methylation signatures through the lens of genomic imprinting

**DOI:** 10.1038/s41598-024-52114-3

**Published:** 2024-01-19

**Authors:** Jean-Noël Hubert, Nathalie Iannuccelli, Cédric Cabau, Eva Jacomet, Yvon Billon, Rémy-Félix Serre, Céline Vandecasteele, Cécile Donnadieu, Julie Demars

**Affiliations:** 1 GenPhySE, Université de Toulouse, INRAE, ENVT, 31326 Castanet-Tolosan, France; 2 Sigenae, GenPhySE, Université de Toulouse, INRAE, ENVT, 31326 Castanet-Tolosan, France; 3 ENVT, 31326 Castanet-Tolosan, France; 4 INRAE, GenESI, 17700 Surgères, France; 5 INRAE, GeT-PlaGe, Genotoul, 31326 Castanet-Tolosan, France; 6 Qualyse, Le Treuil, INRAE, 19000 Tulle, France

**Keywords:** Epigenomics, Animal breeding

## Abstract

Genomic imprinting represents an original model of epigenetic regulation resulting in a parent-of-origin expression. Despite the critical role of imprinted genes in mammalian growth, metabolism and neuronal function, there is no molecular tool specifically targeting them for a systematic evaluation. We show here that enzymatic methyl-seq consistently outperforms the bisulfite-based standard in capturing 165 candidate regions for genomic imprinting in the pig. This highlights the potential for a turnkey, fully customizable and reliable capture tool of genomic regions regulated by cytosine methylation in any population of interest. For the field of genomic imprinting, it opens up the possibility of detecting multilocus imprinting variations across the genome, with implications for basic research, agrigenomics and clinical practice.

## Introduction

Genomic imprinting (GI) is an original molecular phenomenon mediated by the apposition of epigenetic marks (DNA methylation and/or histone marks) leading to allele-specific expression dependent on the parental origin^1^. GI studies intersect with a broad range of biological fields, including evolution biology, developmental biology, molecular genetics and epigenetics. GI is involved in many phenotypes in humans but also contributes to the variability of major agronomic phenotypes^2,3^. Imprinted genes are therefore highly attractive targets and biomarkers^4,5^, which are found isolated or as clusters across the genome, representing 1% to 2% of the total gene content in the best studied mammals. Parent-of-origin (PofO) expression is primarily controlled by differentially methylated regions (DMRs) in a parental way as well^1^. Although knowledge about GI has significantly advanced so far, some technological bottlenecks remain to tackle challenging scientific insights.

To assess whether and how GI is involved in the variability of complex phenotypes, it is critical to *(i)* map and characterize imprinted loci across the genome and *(ii)* identify simultaneously the parental origin of alleles and their methylation status. Rigorously characterizing imprintomes would require the combination of experimental designs such as reciprocal crosses^6^ with whole-genome sequencing technologies^7,8^. However, such cost-consuming methods could not be used as routine molecular tools. In addition, whole-genome approaches would produce a large majority of unnecessary reads for detecting GI since imprinted genes and their associated DMRs represent a small fraction of the genome. Conversely, cheaper sequencing-based methylation profiling methods^9^ would produce insufficient resolution or an incomplete picture of known imprinted regions. Here, we optimized and compared capture-based methylation sequencing technologies aiming for an exhaustive detection of evolutionary conserved imprinted loci across the genome.

We performed our study in the pig (*Sus scrofa*). The pig is particularly attractive as a major species for the improvement of complex phenotypes^10^ and for its epigenomic features different from those of rodents and sometimes similar to those of humans, whose early epigenomic landscape is very specific^11^. More generally, GI research in the pig contributes to advancing the characterization of the porcine genome, which could in the future provide pathophysiology models of human imprinted disorders^12^. We propose here in the pig the first global evaluation of methylation patterns related to genomic imprinting in livestock.

**Figure 1:**
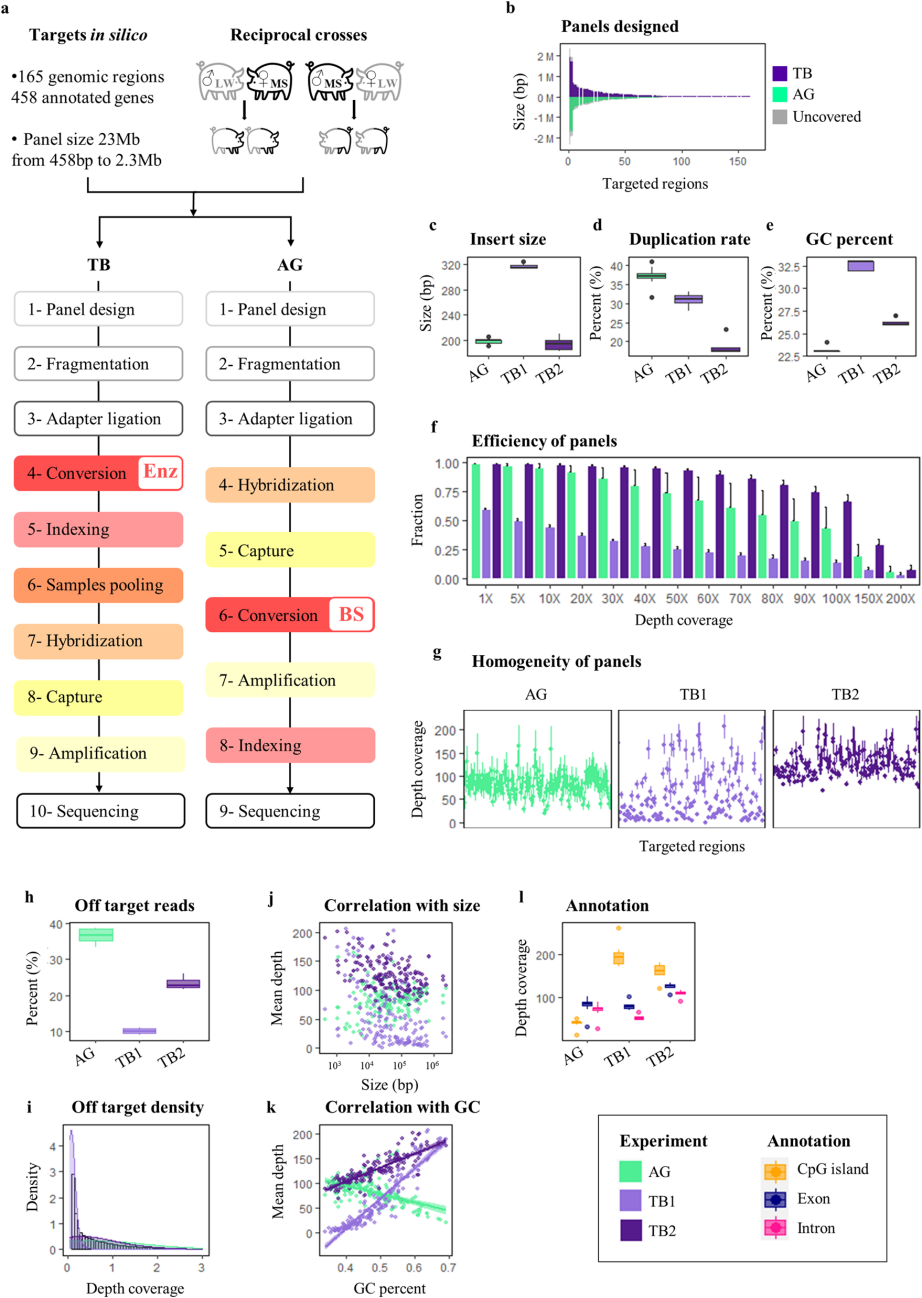
Strategy and performances of technologies. (**a**) Schematic overview of the strategy, including the selection of 165 candidate regions for GI in the pig based on knowledge from humans and mice^1,13^, the use of a reciprocal cross (n = 8) to ensure the determination of parental inheritance^6^ and the tested technologies, Twist Bioscience (TB) *vs*. Agilent (AG). (**b**) Distribution and size of final designed panels by the two manufacturers, AG (green), TB (purple), and uncovered regions (grey). (**c, d, e**) Sequencing performances by technology, including insert size (**c**), duplication rate (**d**) and GC percentage (**e**). (**f**, **g**, **h**, **i)** Panel performances by technology, including efficiency, that is represented as the mean + /- standard deviation of the fraction of targets covered at a specific depth (**f**), homogeneity, that is represented as the mean + /- standard deviation of depth coverage for the 165 targeted regions (**g**), specificity, that is represented as percentage (**h**) and density (**i**) of off-target reads, which mapped outside of the 165 targeted regions. **j**, **k**, Correlation of the mean coverage with either the size (**j**) or the GC percentage (**k**) of the 165 targeted regions. For **c** to **k**, the AG classical protocol is in green and the two TB protocols (TB1 and TB2) are in light and dark purple. **l**, Feature annotation of region per technology.

The strategy implemented below may be applied to any other species with its own custom capture. We *(i)* selected 165 regions in the pig genome based on human and mouse orthologies^1,13^ (https://www.geneimprint.com and https://corpapp.otago.ac.nz/gene-catalogue), since GI mechanisms are quite well conserved in mammals^14^, *(ii)* exploited reciprocal crosses to identify PofO methylation^6^ and *(iii)* tested two different technologies, the novel enzymatic-based Twist NGS Bioscience Methylation Detection System (TB), with two protocols (called TB1 and TB2), and the widely used bisulfite-based Agilent SureSelect Custom DNA Target Enrichment Probes (AG) (Fig. 1a and Extended Data Table s1)^15^.

## Results and discussion

The final designed panels from both technologies covered all the 165 targeted regions but differed slightly in size, with 20.5 Mb and 19.7 Mb for TB and AG technologies, respectively (Fig. 1b and Extended Data Table s1). Sequencing quality analysis showed lower duplication rate and higher GC percentage for TB technology in addition to insert size as expected (Fig. 1c-e and Extended Data Fig. s1a-h). Both target capture efficiency and homogeneity of panels are comparable between AG and TB after optimizing the latter, reaching excellent levels (Fig. 1f and g). Specificity is however more favourable in TB, with much less off-target capture than in AG (Fig. 1h and i and Extended Data Fig. s1g-i). Regarding methylation evaluation and conversion, the enzymatic-based TB technology yielded higher numbers of total and methylated CpGs, as well as less non-CpG methylation than the standard bisulfite-based AG technology (Extended Data Fig. s1j-o). These observations demonstrate an improved specificity of the enzymatic treatment for cytosine conversion and for capturing GC-rich regions such as CpG islands, which represent problematic sources of bias in bisulfite-based sequencing protocols^16^, independently of region size (Fig. 1j-l). Thus, the application of the novel TB approach to GI suggests it outperforms the current technological standard for methylation quantification^15^ (Extended Data Table 2).

**Figure 2:**
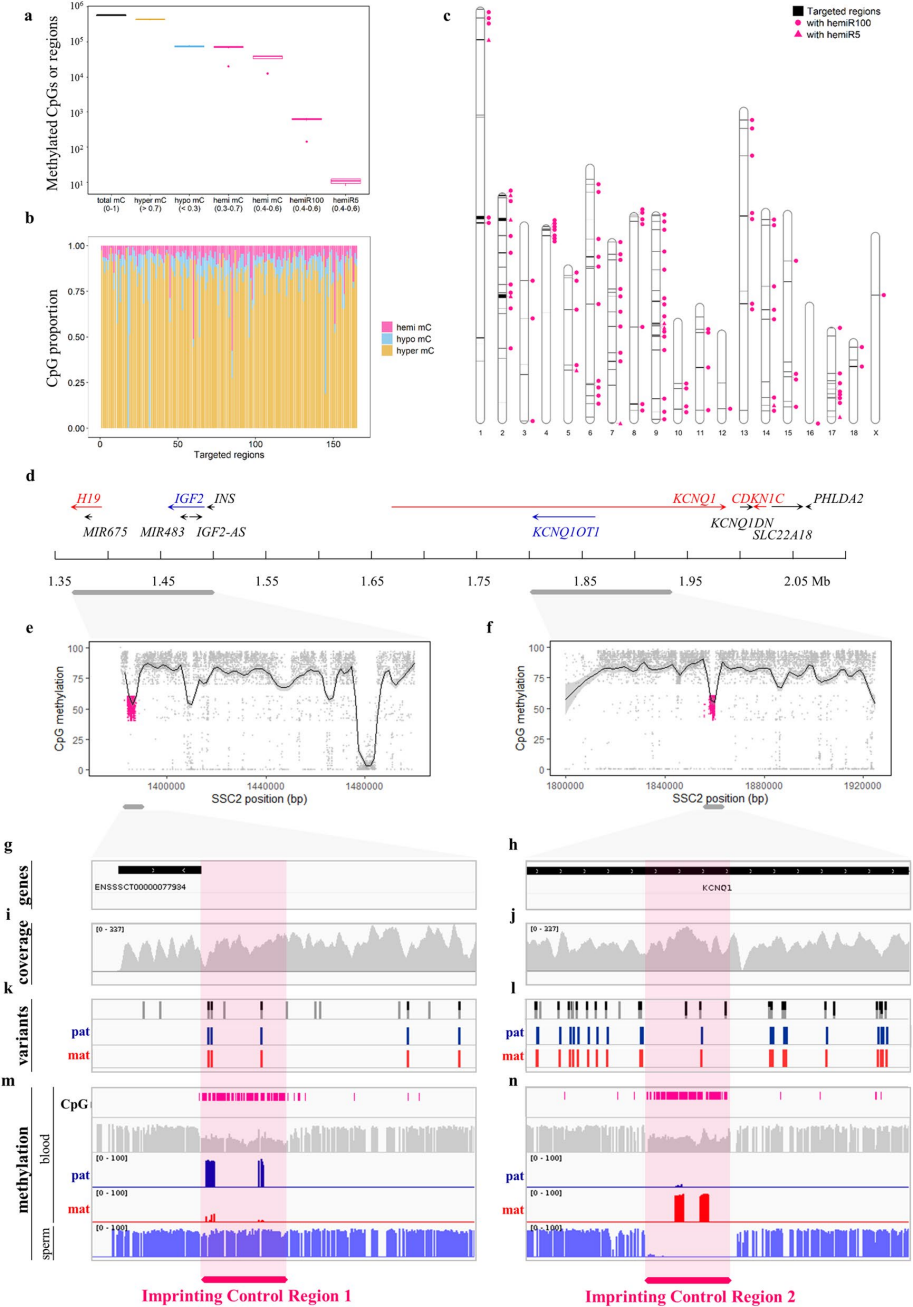
Hemi-methylated CpGs, regions and PofO methylation. Results showed here come from the TB2 protocol. (**a)** Detection, methylation and classification of CpGs. The methylation at CpGs was considered hyper/hypo/hemi when methylation was < 70%, > 30% and between 30–70% and 40–60%, respectively. (**b**) Repartition of hyper/hypo/hemi-methylated CpGs in the 165 candidate regions for GI. (**c**) Location of the hemi-methylated candidate regions across the pig genome. (**d**) Schematic representation of the *IGF2-H19*/*KCNQ1-CDKN1C* imprinted region located on the swine chromosome 2 with genes expressed from the paternal and maternal allele in blue and red, respectively. (**e**, **f**) Magnification of two regions where two clusters of hemi-methylated CpGs, DMRs (pink), were detected. Locally weighted running lines smoother (LOESS) were represented. (**g** to **n**), Screenshots from IGV browser (https://software.broadinstitute.org/software/igv/) magnified in DMRs. (**g**, **h**) Annotation of the pig genome using Sus_scrofa.Sscrofa11.1.104.gtf showing that *KCNQ1OT1* was missing. (**i**, **j**) Coverage. (**k**, **l**) Variants identification and informativity with parental origin in the offspring of reciprocal crosses. (**n**, **m)** Methylation evaluation in blood and sperm tissues and detection of PofO methylation. hemiR100: occurrence of ≥ 5 hemi-methylated CpGs within 100 bp; hemiR5: occurrence of ≥ 5 consecutive hemi-methylated CpGs.

Imprinted genes are regulated by CpG methylation through parental DMRs^1,17^. Such hemi-methylated regions, expected to be methylated on one allele resulting in approximately 50% of methylation, are either somatic or germinal^18^. Such specific DNA methylation patterns belong to the about 2% of CpG exhibiting intermediate DNA methylation values, including parental DMRs, as well as allele- and strand-specific DNA methylation and stochastic DNA methylation^19^. Here, we identified approximately 38,000 hemi-methylated CpGs per individual, clustered in at least 600 hemi-methylated regions fulfilling stringent criteria that are distributed in 123 out of the 165 candidate regions for GI (Fig. 2a–c). Interestingly, the *IGF2-H19*/*KCNQ1-CDKN1C* region, carrying a mutation affecting muscularity in pigs^20^ and hosting some of the best-characterized Imprinting Control Regions (ICRs) in humans and mice^21^, is the top candidate after scanning for GI methylation patterns. Two clusters with more than 100 hemi-methylated CpGs were detected in the region. The first one is located upstream of the 5’ UTR of *H19* and the second one is located upstream of the 5’UTR of *KCNQ1OT1* that is not annotated in the pig reference genome (Fig. 2e–h).

Our strategy relies on next generation sequencing technology that allows the detection of genotypes and CpG methylation simultaneously. Reciprocal crosses were used to phase variants and determine unambiguously the parental inheritance of alleles (Fig. 2i–l and Extended Fig. s2a). We demonstrated, in blood, the paternal specific methylation for the DMR located upstream of the 5’ UTR of *H19* and the maternal specific methylation for the DMR located upstream of the 5’ UTR of *KCNQ1OT1* (Fig. 2m and n and Extended Fig. s2a-c). This result was confirmed on a sperm sample in which the first region was totally methylated while the second one was totally unmethylated (Fig. 2m and n). Both germline DMRs showed similar properties than ICR1 and ICR2, which are known to regulate in humans and mice the *IGF2-H19* and *KCNQ1-CDKN1C* imprinted domains, respectively^1,22,23^.

Altogether, we demonstrated and harnessed the potential of enzymatic methyl-seq to provide a molecular tool adapted to the specific needs of GI. Such a novel tool especially allows detecting PofO methylation, which paves the way to the systematic and routine evaluation of the contribution of GI in both the variability of livestock complex phenotypes^5^ and the diagnosis of human imprinting disorders ^2,7^.

## Materials and methods

### Animals and samples

The study included 10 pigs, 8 pigs were bred at the INRAE experimental farm (https://doi.org/10.15454/1.5572415481185847E12) and 2 pigs come from breeding organizations in accordance with the French and European legislation on animal welfare. The animals belong to the same family, except for one LW animal. Animals were produced in a reciprocal cross design between Large White and Meishan pig breeds.

Ten biological samples were used in the experiment. Nine of them are blood samples collected on EDTA and were stored frozen nine months at − 20 °C. One biological sample is a sperm sample from dose for artificial insemination and was stored two years at − 20 °C. Biological samples were collected at adult developmental stage for all the parents (n = 5) of the reciprocal cross design while biological samples were collected at 1d after birth for all offspring (n = 5) of the reciprocal cross design.

Genomic DNA was extracted from blood using the Genomic-tip 100 DNA kit (Qiagen, 10,243) or using MagAttract HMW DNA kit (Qiagen, 67,563) following manufacturer’s instructions. Genomic DNA was extracted from sperm using standard phenol/chloroform method. DNA purity was determined using the Nanodrop 8000 spectrophotometer (Thermo Fisher Scientific). DNA concentration was determined using the DS DNA Broad Range Assay kit (Invitrogen, ThermoFisher Scientific, Q32850) and was measured with the Qubit3 fluorometer (Invitrogen, ThermoFisher Scientific).

All the procedures and guidelines for animal care were approved by the local ethical committee in animal experimentation (Poitou–Charentes) and the French Ministry of Higher Education and Scientific Research (authorizations n°2,018,021,912,005,794 and n°11,789–2,017,101,117,033,530). All animal and sample information is available at the European Nucleotide Archive (ENA) as accession number PRJEB58558.

### Panel design

Candidate regions for GI in the pig (*Sus scrofa*) were selected based on various publications available in humans and mice^1,13^ and on two databases (https://www.geneimprint.com and https://corpapp.otago.ac.nz/gene-catalogue). Sequences not annotated in the pig genome were subjected to BLAST searches against the Sscrofa11.1 reference. A total of 165 regions ranging from 458 bp to 2.3 Mb, distributed across the 18 autosomes, the X chromosome and 4 scaffolds of the pig reference, were selected. These genomic regions, targeting a total of 23 Mb, were submitted to the two commercial platforms, TB and AG. Each platform used its own confidential algorithm for panel design. The sizes of custom panels from TB and AG were 20.5 Mb and 19.7 Mb, respectively, with all the 165 candidate regions for GI represented.

### Library preparation

The final optimized protocol has been deposited to Protocol Exchange open repository (https://doi.org/10.21203/rs.3.pex-2159/v1). Two types of libraries were generated using AG or TB technology, the latter involving two experiments (TB1 and TB2). The AG and the TB1 experiments were performed at the GeT-PlaGe core facility at INRAE Toulouse (https://doi.org/10.15454/1.5572370921303193E12). The TB2 experiment was performed by Twist Bioscience company (Twist Bioscience, USA).

#### Library preparation and target enrichment with Agilent SureSelect Custom DNA Target Enrichment Probes

Eight library preparations were carried out using the SureSelect Methyl-Seq Target Enrichment kit (Agilent, G9651) following the manufacturer’s protocol (User guide: SureSelect, Agilent Technologies, version E0, April 2018). Genomic DNA (1 µg) was first fragmented using a Covaris M220 focused ultrasonicator in micro-TUBE 50 AFA Fiber screw cap (Covaris, 520,166) for a target insert size of 200 bp under the following conditions: peak power 75W, duty factor 10%, 200 cycles/bursts, 375 s, 8 °C. An additional 0.8X AMPure beads purification step was done to eliminate adaptor dimers.

#### Library preparation and target enrichment with Twist Bioscience NGS methylation detection system

Sixteen library preparations were carried out using an in-house combination of two protocols: NEB-Next Enzymatic Methyl-seq Library Preparation and Twist Bioscience Targeted Methylation Sequencing, using a methyl custom panel. The whole detailed and optimized protocol has been deposited to Protocol Exchange open repository (https://doi.org/10.21203/rs.3.pex-2159/v1). Briefly, eight library preparations were carried out with a first similar development protocol (TB1) in which some adjustments have not yet been made. Differences between protocol^TB1^ and protocol^TB2^ are referenced in the procedure deposited in Protocol Exchange. All library quantifications were performed on a Qubit 3.0 fluorometer with High Sensitivity DNA Quantitation Assay kit according manufacturer’s recommendations (Agilent, ThermoFisher Scientific, Q32851). All library validations were performed on a 2100 Bioanalyzer with High Sensitivity DNA kit according to manufacturer's recommendations (Agilent Technologies, 5067–4626).

### Sequencing

All libraries were quantified by qPCR on QuantStudio 6 device (Applied Biosystems, ThermoFisher Scientific), using the Kapa Library Quantification Kit (Roche, KK4824). Agilent libraries and experiment TB1 libraries were each sequenced on one lane of an Illumina SP NovaSeq 6000 flow cell, using the SP Reagent kit v1.5 300 cycles (Illumina, 20,028,400), according to the manufacturer's recommendations. The loading concentration was 2 nM 25% phiX. Experiment TB2 libraries were sequenced on Illumina P2 NextSeq 2000 flow cell, using the SP Reagent kit v3 300 cycles (Illumina, 20,046,813), according to the manufacturer's recommendations. The loading concentration was 1000 pM 5% phiX. All sequences are available at ENA under study accession PRJEB58558.

### Methyl-seq data analysis

Analyses were performed using the genotoul bioinformatics platform Toulouse Occitanie (Bioinfo Genotoul, https://doi.org/10.15454/1.5572369328961167E12). Methyl-seq reads were processed with the nf-core/methylseq (v1.5) pipeline^24,25^ (https://nf-co.re/methylseq), using the Sscrofa11.1 pig reference and the Bismark^26^ workflow with standard parameters. Sequencing quality analysis was performed with custom Python scripts for comparing AG and TB experiments. CpG calls from TB2 experiment with depth ≥ 20X were further processed with CGmapTools^27^ and inbuilt Linux commands. Cytosines with methylation levels either < 0.3 or > 0.7 were classified as either hypo-methylated or hyper-methylated, respectively. Cytosines with methylation levels between 0.4 and 0.6, indicating potential PofO methylation, were classified as hemi-methylated. This subset of hemi-methylated CpGs was scanned using a sliding window approach with a custom R function to identify hemi-methylated regions potentially compatible with GI. The occurrence of ≥ 5 hemi-methylated CpGs within 100 bp was labelled as hemiR100. A subset of hemiR100, that is the occurrence of ≥ 5 consecutive hemi-methylated CpGs, was made distinct and labelled as hemiR5. Such cutoffs on CpGs-related parameters such as depth, methylation levels and density aim to define hemi-methylated regions incorporating some of the most stringent criteria for targeting epigenetic signatures of GI from reference imprintome studies^7,28^. Neighbouring hemi-methylated regions at a distance less than their initial definition criterion (i.e., 100 bp for hemiR100 and 5 bp for hemiR5) were merged in a single larger region. Top hemi-methylated regions were visually inspected using Integrative Genomics Viewer^29^, identifying when possible the parental origin of methylation in the progeny of the reciprocal cross. A complete list of software versions used in this study is provided in the next section.

### Software used

BEDtools (v2.27.1) ^30^

Bismark (v0.22.3) ^26^

CGmapTools (v0.1.2) ^27^

Cutadapt (v2.9)^31^

nf-core/methylseq (v1.5)^24,25^

Nextflow (v20.01.0)^32^

FastQC (v0.11.9, https://www.bioinformatics.babraham.ac.uk/projects/fastqc/).

Integrative Genome Viewer (v2.8.13)^29^

MultiQC (v1.8)^33^

Qualimap (v2.2.2-dev)^34^

Preseq (v2.0.3)^35^

R base (v4.1.1) with dplyr (v1.0.9), ggplot2 (v3.3.6), RIdeogram (v0.2.2), scales (v1.2.1) and tidyr (v1.2) packages (https://cran.r-project.org/).

Samtools (v1.9)^36^

Trim Galore! (v0.6.4_dev, https://www.bioinformatics.babraham.ac.uk/projects/trim_galore/).

HISAT2 (v2.2.0)^37^

### Supplementary Information

Supplementary Information 1.

Supplementary Information 2.

Supplementary Information 3.

Supplementary Information 4.

## Data Availability

The dataset generated during the current study is available in the ENA data repository (https://www.ebi.ac.uk/ena/browser/home, accession number PRJEB58558).
