# Supplementary figures and images for: Detection of DNA methylation signatures through the lens of genomic imprinting
Source: Sci Rep. 2024 Jan 19;14:1694. doi: 10.1038/s41598-024-52114-3 (PMC10798973; doi:10.1038/s41598-024-52114-3)

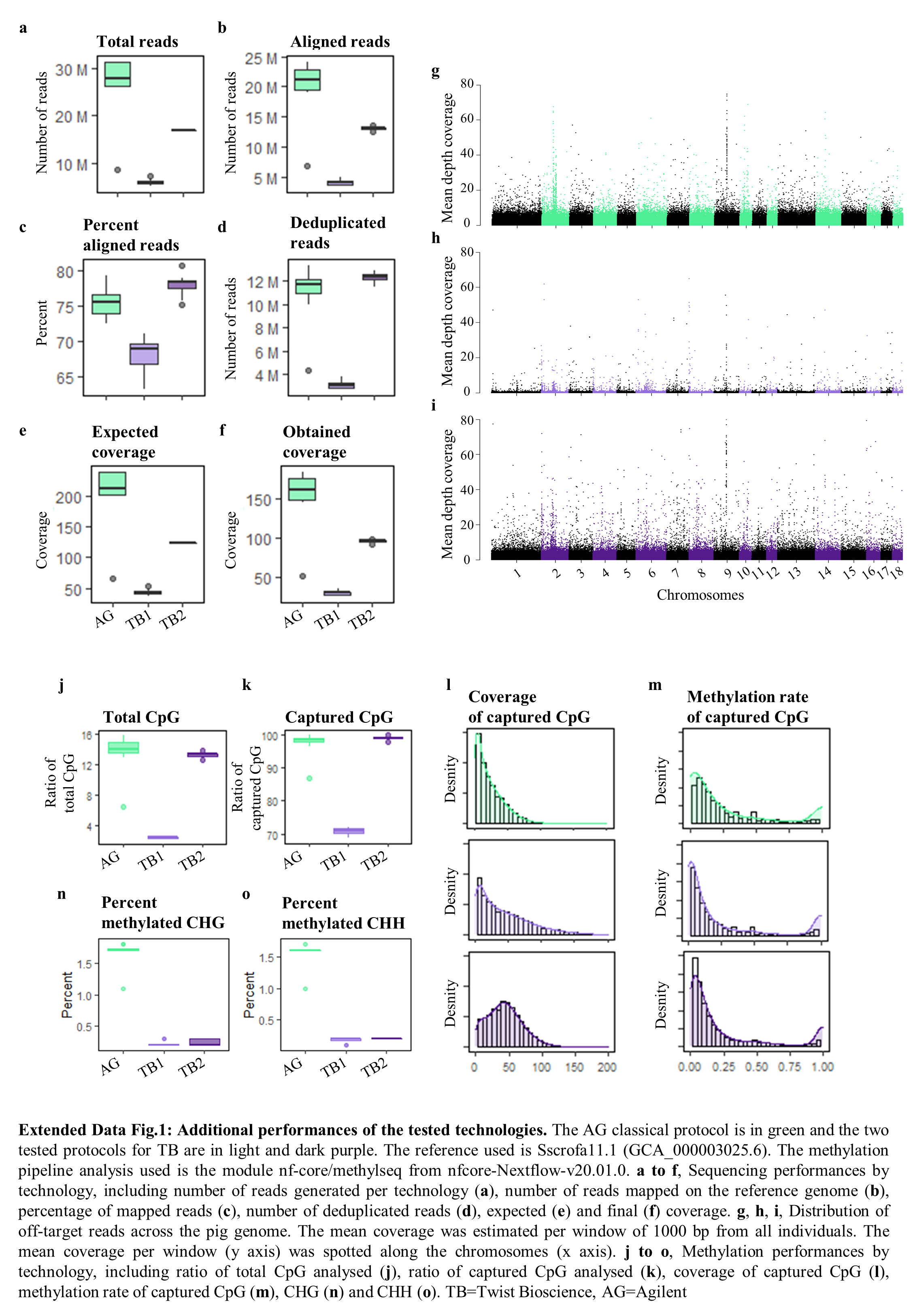

Supplement: Supplementary file 1 — Supplementary Information 1. [file 41598_2024_52114_MOESM1_ESM.png]

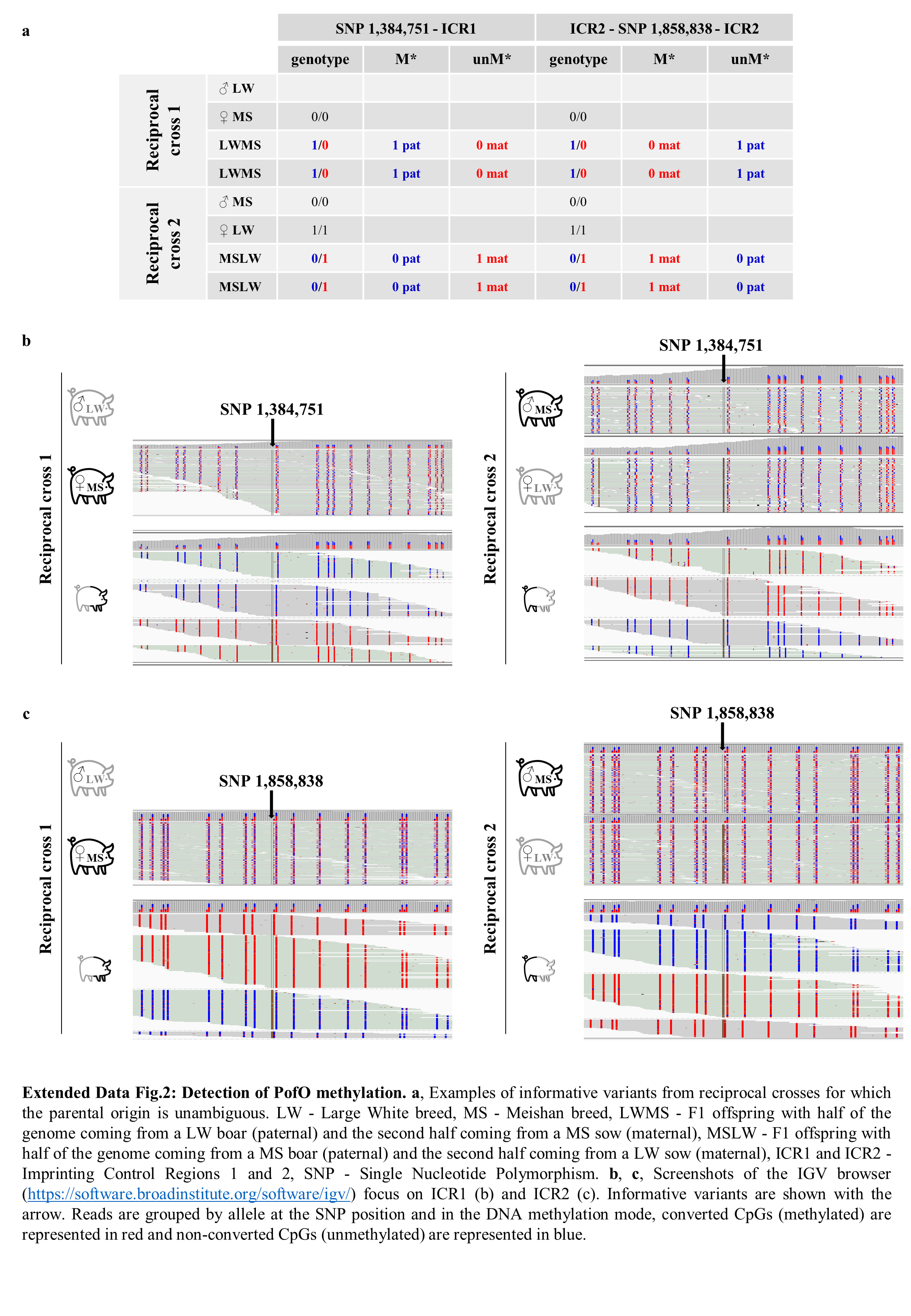

Supplement: Supplementary file 2 — Supplementary Information 2. [file 41598_2024_52114_MOESM2_ESM.png]

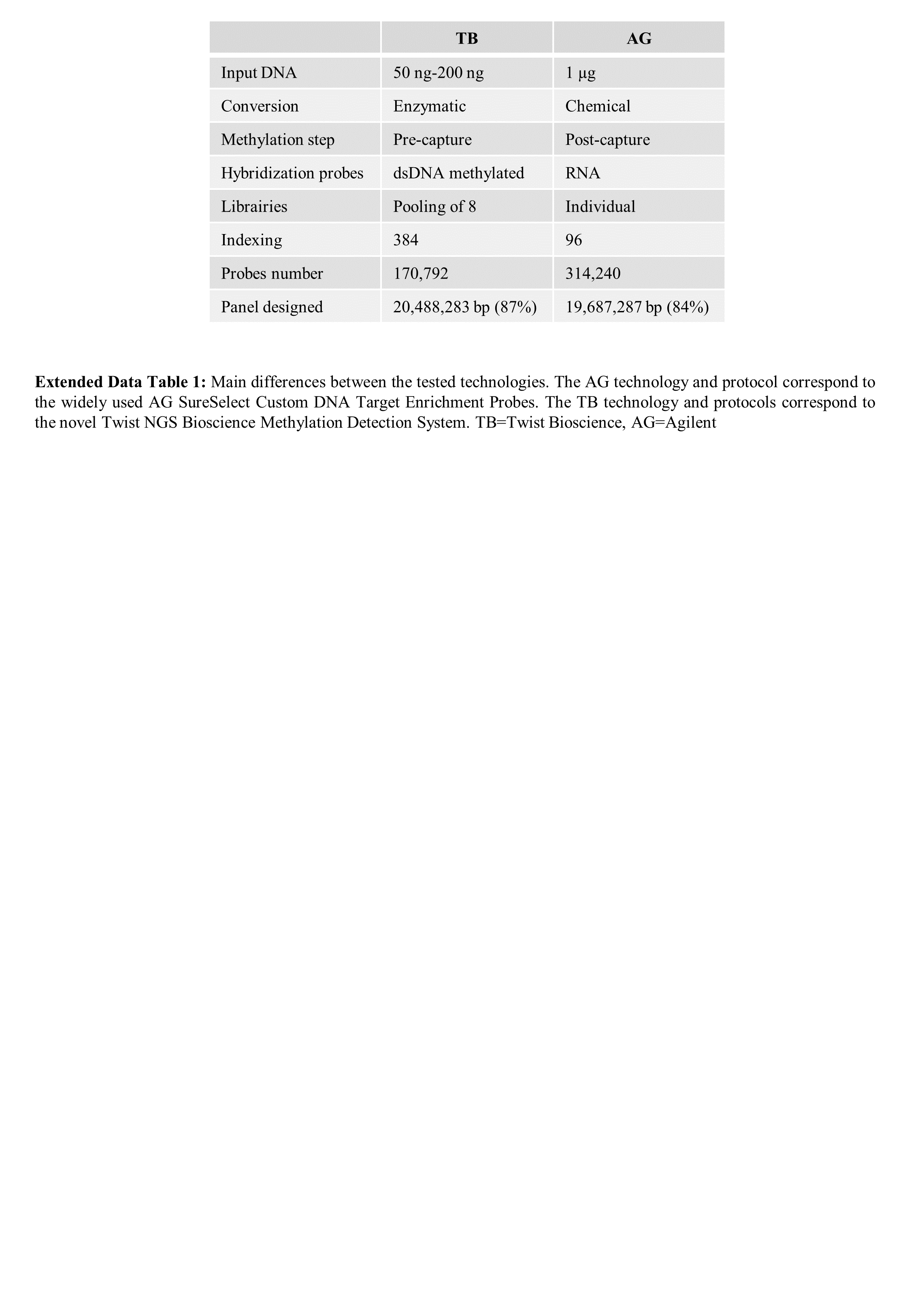

Supplement: Supplementary file 3 — Supplementary Information 3. [file 41598_2024_52114_MOESM3_ESM.png]

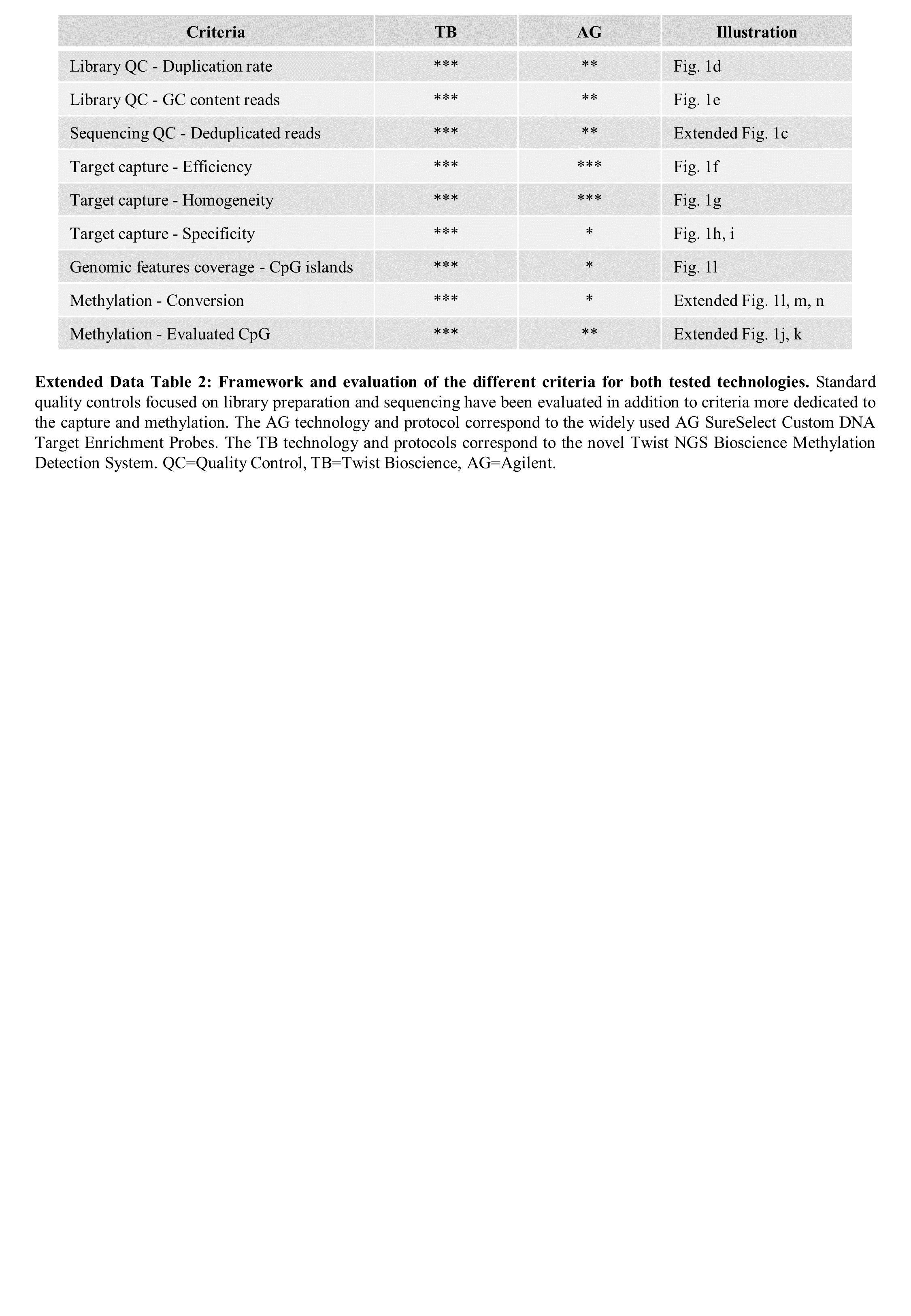

Supplement: Supplementary file 4 — Supplementary Information 4. [file 41598_2024_52114_MOESM4_ESM.png]
